# Symbiotic Nitrogen Fixation and the Challenges to Its Extension to Nonlegumes

**DOI:** 10.1128/AEM.01055-16

**Published:** 2016-06-13

**Authors:** Florence Mus, Matthew B. Crook, Kevin Garcia, Amaya Garcia Costas, Barney A. Geddes, Evangelia D. Kouri, Ponraj Paramasivan, Min-Hyung Ryu, Giles E. D. Oldroyd, Philip S. Poole, Michael K. Udvardi, Christopher A. Voigt, Jean-Michel Ané, John W. Peters

**Affiliations:** a Department of Chemistry and Biochemistry, Montana State University, Bozeman, Montana, USA; b Department of Bacteriology, University of Wisconsin—Madison, Madison, Wisconsin, USA; c Department of Plant Sciences, University of Oxford, Oxford, United Kingdom; d Plant Biology Division, The Samuel Roberts Noble Foundation, Ardmore, Oklahoma, USA; e John Innes Centre, Norwich Research Park, Norwich, United Kingdom; f Department of Biological Engineering, Massachusetts Institute of Technology, Cambridge, Massachusetts, USA; North Carolina State University

## Abstract

Access to fixed or available forms of nitrogen limits the productivity of crop plants and thus food production. Nitrogenous fertilizer production currently represents a significant expense for the efficient growth of various crops in the developed world. There are significant potential gains to be had from reducing dependence on nitrogenous fertilizers in agriculture in the developed world and in developing countries, and there is significant interest in research on biological nitrogen fixation and prospects for increasing its importance in an agricultural setting. Biological nitrogen fixation is the conversion of atmospheric N_2_ to NH_3_, a form that can be used by plants. However, the process is restricted to bacteria and archaea and does not occur in eukaryotes. Symbiotic nitrogen fixation is part of a mutualistic relationship in which plants provide a niche and fixed carbon to bacteria in exchange for fixed nitrogen. This process is restricted mainly to legumes in agricultural systems, and there is considerable interest in exploring whether similar symbioses can be developed in nonlegumes, which produce the bulk of human food. We are at a juncture at which the fundamental understanding of biological nitrogen fixation has matured to a level that we can think about engineering symbiotic relationships using synthetic biology approaches. This minireview highlights the fundamental advances in our understanding of biological nitrogen fixation in the context of a blueprint for expanding symbiotic nitrogen fixation to a greater diversity of crop plants through synthetic biology.

## INTRODUCTION

There is growing interest in increasing the contribution of biological nitrogen fixation to the growth of crop plants in agriculture. Symbiotic nitrogen fixation is largely limited to legumes in agricultural systems, but there are a number of microorganisms, including some diazotrophs, that inhabit the rhizosphere of other crop plants, some of which have been shown to enhance plant growth. Here, we present an overview of the diversity and specificities of associations between diazotrophs and their host plants and the biology and biochemistry of these nitrogen-fixing symbiotic associations. Understanding plant and microbe mechanisms involved in the formation and functions of these symbioses to solve the nitrogen fixation problem will position us to engineer these processes into nonfixing food crops, such as cereals and agriculturally important eudicots. Initial challenges include identifying a suitable microbial partner, initiating intracellular accommodation, controlling the plant microbiome, and keeping cheaters under control. We discuss perspectives and limitations to engineering a nitrogen-fixing ability in plants based on knowledge of symbiotic nitrogen fixation in legumes and nonlegumes.

## SYMBIOTIC NITROGEN FIXATION

### Diversity of nitrogen-fixing plant-microbe associations.

Nitrogen-fixing bacteria are found in several phyla (1), and representatives from most (if not all) of these phyla are known to engage in nitrogen-fixing symbiosis with plants (2). Reciprocally, plants have developed multiple solutions to associate with and accommodate diazotrophs in order to acquire atmospheric nitrogen. Proximity between a bacterial symbiont and plant host is a key element for nutrient exchanges between them and falls into three broad categories, based on the degree of intimacy and interdependency of the plant and microbe: loose associations with free-living nitrogen fixers, intercellular endophytic associations, and endosymbioses.

**FIG 1:**
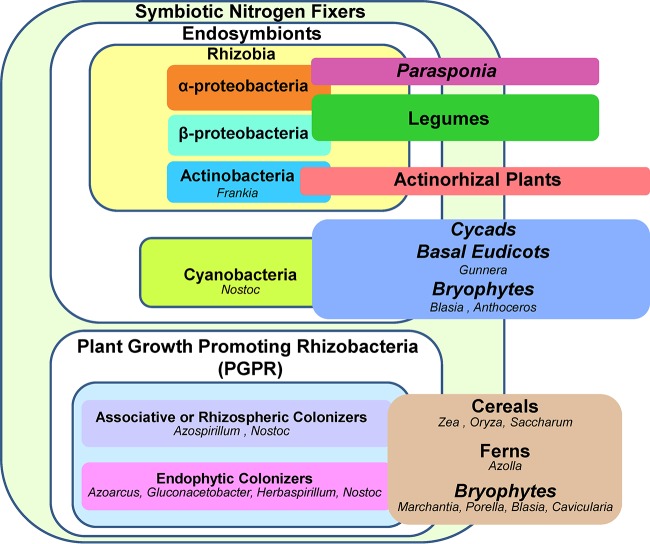
Schematic representation of the different associations between diazotrophs and plant hosts. Diazotrophs are divided in two main groups: root-nodule bacteria and plant growth-promoting rhizobacteria (PGPR). Root-nodule bacteria include rhizobia and Frankia. Rhizobia (alpha- and betaproteobacteria) enter into a symbiotic association with legumes and Frankia with actinorhizal plants. Alphaproteobacteria can also nodulate Parasponia species. Some plants develop endosymbiotic interactions with nitrogen-fixing cyanobacteria (Nostoc). PGPRs include proteobacteria (alpha-, beta-, and gammaproteobacteria), actinobacteria, bacilli, and cyanobacteria. Many PGPRs develop associative or endophytic associations with cereals. Some cyanobacteria found within plant tissues are classified as endophytes.

Interactions between plants and associative nitrogen-fixing bacteria, which are considered a subset of plant growth-promoting rhizobacteria (PGPR) (Fig. 1), are the simplest form of nitrogen-fixing symbiosis. These associative bacteria respond to root exudates via chemotaxis to, and colonization of, the rhizosphere of many plants but typically do not invade plant tissues (3, 4). Nitrogen-fixing PGPR have been identified among the bacilli and especially among the proteobacteria (5). Their proximity to the root enables them to impact plant resource acquisition (nitrogen, phosphorus, and essential minerals), yield, and growth (6). Some of the best-studied species of associative PGPR belong to the genus Azospirillum, which are able to improve the fitness of several crops, including wheat, maize, and rice (7). Azolla ferns, which have been used as companion plants in rice agriculture for centuries, accommodate the heterocystous cyanobacterium Nostoc azollae (formerly Anabaena azollae) within specialized leaf cavities (8).

Many species of diazotrophic bacteria have evolved beyond surface colonization to spread and multiply within plant tissues without causing damage and eliciting significant defense reactions. These bacteria, such as Azoarcus, Herbaspirillum, and Gluconacetobacter (Fig. 1), are classified as endophytes due to their tight association with plant tissues (9). Bacterial endophytes are ubiquitous and have been isolated from surface-sterilized tissue from almost all plants examined to date (10). Their association can be obligate or facultative, and they exhibit complex interactions with their hosts that range from mutualism to parasitism. They typically enter plant tissues through natural openings (stomata) or through cracks at the site of lateral root emergence, for instance (11). Research on bacterial endophytes has mainly focused on quantifying the amount of nitrogen fixed and on identification of the diazotrophs; consequently, very little is known about the molecular mechanisms involved in forming and maintaining the cooperation. Cyanobacteria are also frequently found within plant tissues. Nostoc is endophytic with two genera of liverworts (Blasia and Cavicularia) and all hornworts. Colonization can take place in dome-shaped auricles on the thallus of liverworts or in slime cavities of the thallus or mucilage-filled canals that run parallel to the thallus of hornworts (12). Nostoc is also able to endophytically colonize coralloid roots of cycads. The mechanism of recruitment is unknown, but the cyanobacteria are found embedded in mucilage in a specific cortical layer of the coralloid root between elongated specialized cells (13).

The most elaborate form of nitrogen-fixing plant microbe association is endosymbiosis. Bacterial endosymbionts are generally acquired from the environment and are accommodated inside plant cells within plant-derived membranes. Some plants interact with nitrogen-fixing cyanobacteria. In the symbiosis between plants of the genus Gunnera and cyanobacteria of the genus Nostoc, seedlings recruit the endosymbiont by secretion of carbohydrate-rich mucilage. Nostoc subsequently enters through specialized glands and then is accommodated within cells of the inner cortex. Filaments of Nostoc are surrounded by the host's plasma membrane, which acts as the interface for nutrient exchange (14). The most well-studied plant endosymbioses are those between actinorhizal plants and Frankia bacteria and between legumes and rhizobia, which we will discuss in more depth below.

### Signaling, infection, and specificity.

The establishment and functioning of an effective symbiosis is dependent on genetic determinants in both plant and bacteria. The fully compatible symbiosis proceeds from recognition, penetration, stimulation of host cell division, and differentiation of the endosymbiont.

## ENDOSYMBIONT

Legume-Rhizobium symbiosis starts with a molecular dialogue between the two partners. The legume secrete a cocktail of phenolic molecules, predominantly flavonoids and isoflavonoids, into the rhizosphere. These signals are taken up by rhizobia, bind the transcriptional regulator NodD, and activate a suite of bacterial nodulation genes (15). These nodulation genes are responsible for the production of lipochitooligosaccharides (LCOs) called Nod factors. Nod factors are key symbiotic signals and are indispensable in the specific host-Rhizobium interaction and at later stages in the infection process and nodule organogenesis (16). Nod factors are active at very low concentrations (nanomolar to picomolar range). Nod factors from different rhizobia share the same chitin-like *N*-acetylglucosamine oligosaccharide backbone with a fatty acyl chain at the nonreducing end, but they differ in the length of their backbone, size and saturation of the fatty acyl chain, and have additional modifications at either end, such as glycosylation and sulfation. Such decorations on the ends of LCOs play a crucial role in determining whether the Nod factors can be perceived by a specific host (15). The perception of Nod factor signals in legumes is mediated by Nod factor receptors (NFRs), which are LysM domain receptor kinases. It has been demonstrated by genetic and molecular analyses in pea, soybean, and Lotus japonicus that NFRs are host determinants of symbiosis specificity (17–19).

Nod factors trigger plant cell division and meristem formation, and the rhizobia infect legume roots through crack entry, intercellular colonization of epidermal cells, or the well-studied formation of infection threads (20). Rhizobia eventually enter root cortical cells via endocytosis, where they differentiate into nitrogen-fixing bacteroids within a unique plant organelle called the symbiosome. The symbiosome is delimited by a plant-derived membrane that controls nutrient exchange between the symbionts. Two main types of nodules are formed on the various legume species, indeterminate or determinate, depending on whether or not the meristem remains active for the life of the nodule, respectively. Both of types of legume nodules have a peripheral vasculature, in contrast to roots (21).

The strategies used by Frankia spp. to infect actinorhizal plants are quite similar to those used by rhizobia. Depending on both the host species and Frankia clade, root hair, crack entry, or an intercellular infection mode is employed (22). Actinorhizal nodules are indeterminate, have a central vasculature (like roots), and fix nitrogen in amounts comparable to legumes. In addition to legumes and actinorhizal plants, Parasponia andersonii (family Cannabaceae) displays a unique nitrogen-fixing symbiosis, as it is the only nonlegume known to be nodulated by rhizobia (23) (Fig. 1). Rhizobia invade Parasponia spp. by crack entry and then proliferate and fix nitrogen within infection threads that ramify throughout the nodule tissue. The rhizobia are never released into cells in symbiosomes, nor do they terminally differentiate. Because Parasponia evolved this ability relatively recently, it has been suggested that it represents a fairly primitive form of nodulation (24).

The degree of specificity between legumes and rhizobia varies. For example, although the Nod factors produced by Rhizobium etli and Rhizobium loti are identical, the two species have distinct host ranges (Phaseolus spp. and Lotus spp., respectively) (25). Furthermore, two rhizobia that nodulate the same plant may secrete different Nod factors. Rhizobium tropici and R. etli produce sulfated and acetylfucosylated Nod factors, respectively, but both effectively nodulate Proteus vulgaris. Likewise, Bradyrhizobium elkanii, Bradyrhizobium japonicum, Rhizobium sp. strain NGR234, and Sinorhizobium fredii strain USDA257 have a number of common hosts, but their Nod factors vary considerably (26). Another class of bacterial components that can interact directly with the host is bacterial surface polysaccharides: exopolysaccharides (succinoglycans and galactoglucans), lipopolysaccharides, capsular polysaccharides, and cyclic β-glucans. They have been reported in numerous studies as being symbiotically important, and depending on the particular system, a defect in surface polysaccharides may cause failure of symbiosis at either an early or late stage (27–32). It was recently reported that some strains of Frankia possess the ability to produce LCOs (33), but the majority of Frankia strains employ an unknown signal that is may be structurally unrelated to LCOs (34).

In Azolla-Nostoc symbiosis, specificity is maintained by vertical inheritance of the cyanobacterium. During sporulation, Nostoc filaments are packaged into sporocarps by sporangial pair hairs and retained until nutrient exchange can be reestablished during embryogenesis. This specificity has been maintained over the course of evolution, with the cyanobacteria cospeciating with the fern (35). In the Gunnera-Nostoc symbiosis, the flow of mucilage excludes most bacteria, and only compatible symbionts achieve intracellular infection. Elements in the mucilage of all Nostoc hosts act as chemoattractants and induce differentiation into specialized motile filaments called hormogonia (12, 14).

## HOST PLANT

All plants release a significant amount of organic carbon into the soil in the form of cell lysates, intact border cells, mucilage, and root exudates (36). The amount and type of exudates depend on plant genotype and growth stage, vary across different environmental conditions (soil type, soil moisture, nutrient availability, or toxicity), and are greatly affected by the organisms living in the rhizosphere. Exudates are complex mixtures of low-molecular-weight organic substances, like sugars, amino and organic acids, fatty acids, sterols, growth factors, and vitamins (37). It is well known that root exudates can influence the soil microbial community structure and biogeochemical cycles of key nutrients, such as nitrogen and phosphorous (38). The composition of exudates is highly varied between plant species and allows the recruitment of unique populations of prokaryotes and eukaryotes (39). Plants can enrich their rhizosphere with specific microbiota by the secretion of particular carbon sources. For example, dicarboxylates in tomato root exudates favor the growth of Pseudomonas biocontrol strains (40, 41). Pea plants select for their symbiont Rhizobium leguminosarum by the excretion of homoserine into the rhizosphere (42, 43). In fact, Rhizobium leguminosarum has been shown to contain a pea-rhizosphere-specific plasmid that is globally upregulated in the pea rhizosphere (44). Root exudates also play an important role in plant defense through the secretion of phytochemicals that can inhibit the growth of certain microbes (45). The ability to tolerate these chemicals can play an important role in the ability to colonize the plant. For example, the PGPR Pseudomonas putida is both tolerant of and attracted by the main antimicrobial benzoxazinoid produced by maize (46). Pseudomonads also possess specialized gene sets that allow them to overcome nonhost isothiocyanate resistance in Arabidopsis (47). Some legumes produce toxic amino acid derivatives (for example, mimosine and canavanine) that are harmful to the general root microbiota but can be resisted or even catabolized by their rhizobial symbionts (48, 49).

Remarkably, some bacteria have the ability to modify the plant rhizosphere to favor their growth or the growth of their siblings. Agrobacterium strains contain genes on their tumor-inducing plasmids that encode the synthesis and catabolism of novel carbon and nitrogen compounds from the condensation of sugars and amino acids. Opine synthesis genes are transferred to the plant host upon invasion and result in the production of opines by the plant that provide a specialized ecological niche that favors the growth of Agrobacterium (50). Some strains of Sinorhizobium meliloti and Rhizobium leguminosarum are capable of synthesizing inositol derivatives called rhizopines during nitrogen fixation in legume nodules (51). The ability to catabolize these compounds has been proposed to provide a competitive advantage to their siblings in the rhizosphere (52).

Transgenic plants expressing opine biosynthesis genes have been generated and shown to reshape rhizosphere populations to increase the population densities of opine-catabolizing bacteria compared to wild-type plants (53, 54). These findings provide proof of principle for the biased rhizosphere concept, bolstered by observations that changes in population density correlated with levels of opine production under a range of concentrations in the two phylogenetically distant plant species Lotus corniculatus and Arabidopsis thaliana (54, 55). Engineering of Pseudomonas to catabolize opines resulted in a competitive advantage for colonization compared to wild-type Pseudomonas during colonization of transgenic opine-producing plant roots (56). Thus, biased rhizospheres and targeted rewards represent an exciting opportunity for engineering to both provide a competitive advantage to a symbiont in the rhizosphere and potentially provide dedicated carbon sources to energize nitrogen fixation.

Plants have evolved several mechanisms for exerting additional control over the symbiont once symbiosis has been established. It has recently emerged that exopolysaccharides on the cell surface may serve as a second checkpoint for appropriate partner selection and are recognized by specific receptors in the plant (32). Nod factors also serve as an important signal to suppress plant immunity and permit the invasion of partner rhizobia.

Once successful invasion of the plant and nodule formation has occurred, there is some evidence that legumes are able to limit the proliferation of “cheater” bacteria that express the traits for successful invasion but not for efficient nitrogen fixation. This process is essential to guarantee the stability of cooperation in these mutualistic associations. It has been established that legumes are able to monitor symbiotic performance and sanction nodules that are ineffective (57). Sanctioning may be accomplished by restricting the supply of sugars to ineffective nodules, such that the plant only dedicates resources to nodules that supply a significant amount of nitrogen in return for the carbon they receive. This leads to premature senescence of nodules harboring low-quality symbionts. It has been proposed that Parasponia spp., some woody legumes, and actinorhizal plants control their symbionts by the production and storage of antimicrobial phenolic compounds in uninfected cells (24). There is also some evidence of control of cheaters in symbiosis with Nostoc. When the global nitrogen cycle regulator *ntrC* of Nostoc is mutated, the host Anthoceros limits the extent of infection (12). Other mutualistic associations, such as the arbuscular mycorrhizal symbiosis, are stabilized through mutual and targeted rewards (58).

Several plant clades have evolved short defensin-like proteins that further control the behavior of the bacterial symbiont. Legumes in the inverted repeat-lacking clade (but not legumes in the related robinioid clade) produce hundreds of small, nodule-specific, and cysteine-rich peptides. These peptides perturb the cell cycle, leading to endoreduplication of both plant and bacterial genomes, disruption of membrane stability, alteration of gene expression, and promotion of terminal differentiation of the Rhizobium species (59). More recently, sets of defensin-like peptides with similar properties have been found in dalbergioid legumes (60) and in three genera of actinorhizal hosts (61).

### Nutrient exchange.

**FIG 2:**
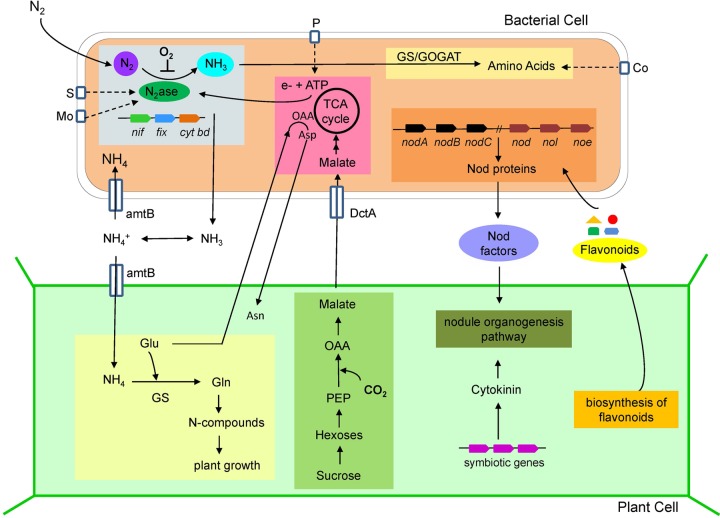
Schematic representation of partnership between a diazotrophic bacterial cell and a nodulating plant cell during symbiotic nitrogen fixation. Rhizobia induce the formation of nodules on legumes using either Nod factor-dependent or Nod factor-independent processes. In the Nod factor-dependent strategy, plants release signals, such as flavonoids, that are perceived by compatible bacteria in the rhizosphere. This activates the nodulation (*nod*) genes of rhizobia, which in turn synthesize and release bacterial signals, mainly lipochitooligosaccharides (LCOs) (Nod factors), which trigger early events in the nodulation process. Synthesis of the Nod factors backbone is controlled by the canonical *nodABC* genes, which are present in all rhizobia, but a combination of other nodulation genes (*nod*, *nol*, or *noe*) encode the addition of various decorations to the core structure. In the Nod factor-independent process, bacteria enter in the plant via cracks in the epidermis. Accumulation of cytokinin synthesized by the bacteria in these infection zones might trigger nodule organogenesis. In the mature nodule, bacteria progressively experience lower oxygen concentrations and differentiate into bacteroids, fixing diffused nitrogen gas using their nitrogenase enzyme complex. NH_3_ produced by nitrogenase from the bacteria (*nif*, *fix*, and cytochrome *bd*) can be incorporated into amino acids via the glutamine synthetase-glutamate synthase (GS-GOGAT) pathway. NH_3_ can also diffuse through the bacterial membrane and be transported to the plant cytoplasm via ammonia transporters (e.g., AmtB), where it is assimilated into nitrogen compounds (amino acids, proteins, and alkaloids) in exchange for food molecules, e.g., glucose, amino acids, and other saccharides. The plant provides amino acids to the bacterial cell and in return the bacterial cell cycles amino acids back to the plant for asparagine synthesis. Other nutrients have to be made available for the microbe, including phosphorus, sulfur, molybdenum, and cobalt. Asn, asparagine; Asp, aspartate; αKG, alpha ketoglutarate; AmtB, ammonia transporter; Co, cobalt; *cyt bd*, cytochrome *bd*; DctA, dicarboxylate transporter; Glu, glutamate; Gln, glutamine; GOGAT, glutamate synthase; GS, glutamine synthetase; HCO_3_^−^, bicarbonate; Mo, molybdenum; NH_3_, ammonia; N_2_ase, nitrogenase; Nod factors, nodulation factors; NFR, Nod factor receptor; OAA, oxaloacetate; P, phosphorus; S, sulfur.

The driving force of symbiosis between a plant and a nitrogen-fixing microorganism is the exchange of nutrients between the two partners. In return for fixed nitrogen, the plant typically provides its bacterial symbiont with a carbon source and, depending on the intimacy of the symbiosis, other crucial nutrients. Both organisms change their metabolic routines in order to accommodate to each other's needs, a process that is monitored and regulated by both partners (Fig. 2).

## EFFICIENT DELIVERY AND UPTAKE OF AN ENERGY SOURCE

**FIG 3:**
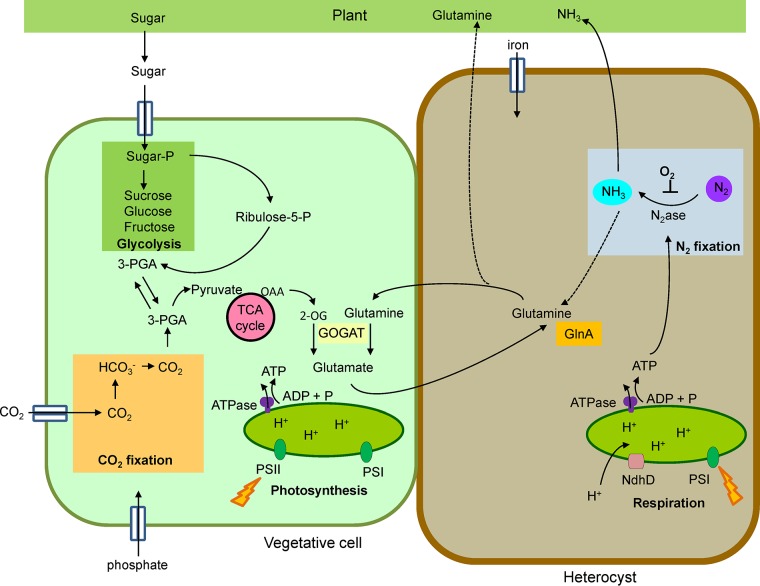
Schematic illustration of important metabolic pathways in associations of nitrogen-fixing cyanobacteria and host plant. The cell on the left represents a vegetative cell, while the cell on the right represents a nitrogen-fixing heterocyst. Important metabolic pathways in associations of nitrogen-fixing cyanobacteria and host plant are glycolysis, carbon fixation, photosynthesis, respiration, and nitrogen fixation. The nitrogen fixed in the heterocyst is incorporated via the GS-GOGAT pathway and used for the synthesis of amino acids, although during symbiosis, most nitrogen is exported to the plant as NH_3_. In exchange, sugars are provided by the host plant. GOGAT, glutamate synthase; GlnA, glutamine synthetase; HCO_3_^−^, bicarbonate; NH_3_, ammonia; N_2_ase, nitrogenase; OAA, oxaloacetate; 3-PGA, polyglycolic acid; PGAL, phosphoglyceraldehyde.

Although the cyanobacterium Nostoc supports nitrogen fixation through photosynthesis under free-living conditions, when associated with a photosynthetic partner, it depends on carbon sources derived from the host (Fig. 3). The main sugars known to support heterotrophic growth are sucrose, glucose, and fructose (14, 62). Likewise, the cyanobacterium Nostoc reduces its carbon fixation to a fraction of what it does under free-living conditions (∼10%), depending on sucrose from the host to make up the difference (8, 12). While it is unknown whether cyanobacteria in tripartite lichens acquire carbon from the phycobiont (either directly or via the mycobiont), cyanobacteria in bipartite lichens must fix their own carbon (63). It is not known whether Nostoc gets organic carbon from its cycad hosts or from its own dark-phase carbon fixation mechanisms (13).

The bacterium Gluconacetobacter is primarily found within both xylem and phloem of its sugarcane host, where it has access to host-produced sucrose (and all its other nutritional requirements). It is unable to transport sucrose, so it secretes enzymes to break down sucrose, and the fructose unit is ultimately used to synthesize the fructooligosaccharides and levan that can then be taken up and utilized (11).

In the symbiosis between Frankia and actinorhizal plants, it is not known which of the carbon compounds derived from sucrose are actually metabolized by Frankia. Experiments performed with vesicle clusters isolated from Alnus nodules have shown that several carbon compounds, including glucose, fructose, sucrose, maltose, dicarboxylic acids, amino acids, succinate, and isocitrate, can be metabolized by symbiotic Frankia. However, it remains unclear which, if any, of these are made available to bacteria within nodules (64).

Inside newly formed legume nodules, rhizobia differentiate and depend on carbon sources derived from the plant to sustain metabolism, including nitrogen fixation (Fig. 2). Plant metabolism is altered to support this energy demand. Genes involved in metabolic pathways, like glycolysis, photosynthesis, amino acid biosynthesis, purine and redox metabolism, and metabolite transport, are all upregulated during symbiosis (65, 66). The primary metabolite is sucrose, which is produced in the aerial parts of plants and travels through phloem to the root nodule, where it is catabolized (67). In nodule cells, sucrose is cleaved reversibly to UDP-glucose and fructose by sucrose synthase and irreversibly to glucose and fructose by invertase (68, 69). Hexoses subsequently enter glycolysis, which is upregulated transcriptionally in the nodules (65), to produce phosphoenolpyruvate, which in turn is converted to dicarboxylic acids. Several studies have shown that carbonic anhydrase, phosphoenolpyruvate carboxylase, and malate dehydrogenase are upregulated during nodule development, which directs carbon flow toward malate (70). The exchange of metabolites between the plant and bacteroids does not happen freely but is facilitated by specialized transporters. Analysis of the genomic inventory of Medicago truncatula transporters revealed that a wide range of transporters are induced during nodule development (66). Among these are genes encoding putative sugar transporters, amino acid transporters, and sulfate transporters (71). In Rhizobium-legume symbiosis, carbon is specifically supplied to the bacteroids in the form of dicarboxylic acids, such as malate (72). After crossing the symbiosome membrane that separates the bacteroids from the plant cell cytoplasm, dicarboxylates are taken up by DctA, a transporter of the major facilitator superfamily (73). Dicarboxylic acids are assimilated by gluconeogenesis or catabolized via enzymes of the tricarboxylic acid (TCA) cycle to provide the reductant and ATP required for nitrogen fixation (74, 75).

## EFFICIENT RELEASE AND UPTAKE OF NITROGEN

Irrespective of the carbon source, the exchange of fixed nitrogen is another nutrient important for the symbiosis to be mutually beneficial. Specifically, bacterial nitrogen metabolism must be altered so that nitrogen is excreted rather than incorporated into the microbial biomass. The plant host appears to directly interfere with bacterial amino acid biosynthesis and thereby force the release of nitrogen (76).

In nitrogen-fixing Rhizobium bacteroids, evidence suggests that nitrogen metabolism is significantly altered during bacteroid differentiation, and ammonia assimilation is effectively shut down (70). Rhizobium leguminosarum bacteroids become symbiotic auxotrophs for branched-chain amino acid transport and become dependent on the plant for the supply of amino acids. Mutants of the branched-chain amino acid ABC transporters Aap and Bra are unable to fix nitrogen for the host plant (76). R. leguminosarum mutants of ammonium assimilation are unaltered in their capacity for symbiotic nitrogen fixation (77, 78). The inactivation of ammonia assimilation in the bacteroid may be accomplished via an unknown and presumably plant-regulated posttranslational modification of the enzyme glutamine synthetase (GS) (79).

In legume-Rhizobium symbiosis, ammonia produced by nitrogenase is delivered to the plant cell as NH_4_^+^ and/or NH_3_ (Fig. 2). Ammonia in its neutral lipophilic form probably crosses the bacteroid membranes via diffusion. The bacterial NH_4_^+^ transporter AmtB, which transports NH_4_^+^ in the opposite direction (i.e., into the bacteroid), is repressed in bacteroids, ensuring that NH_3_ lost from the cell is not recovered by the bacterium but rather is taken into the plant cytoplasm. After entering the symbiosome space between the bacteroid and the symbiosome membrane, ammonia is protonated to NH_4_^+^ because of the acidic environment there (80). In the next step, ammonium crosses the symbiosome membrane and enters the cytoplasm of the infected plant cell, where it is rapidly assimilated into organic form. Two possible pathways exist for ammonium transport across the symbiosome membrane: one through an NH_3_ channel (81), and the other through a cation channel that transports K^+^, Na^+^, and NH_4_^+^ (82). Once inside the plant cell, ammonia is assimilated into amino acids mainly by the action of GS, glutamate synthase (GOGAT), and aspartate aminotransferase. The expression of genes encoding these enzymes is induced during nodule development (65). Interestingly, nodulin 26, which can transport NH_3_ (83), interacts physically with cytosolic GS that is responsible for the assimilation of ammonia to glutamine (84). Several other genes encoding aquaporin-like proteins that potentially transport ammonia are induced in infected cells of Medicago truncatula nodules (71). The symbiosome membrane NH_4_^+^/K^+^ channels have not yet been identified genetically.

In actinorhizal plant-Frankia symbiosis, the bacterial GS remains fully functional, but downstream components of amino acid biosynthesis are downregulated (unlike in legume-Rhizobium symbiosis). Fixed nitrogen is released to the plant in the form of amino acids or amides, with the exact chemical species varying according to the plant host. These are then broken back down to NH_4_^+^, which is then assimilated by the actinorhizal host by the action of GOGAT (65).

In plant-Nostoc symbiosis, up to 80% of the cyanobacterial cells differentiate into heterocysts in order to maximize nitrogen fixation. The percentage of differentiation varies according to the host, with the lowest rates in the associative symbiosis with Azolla (8) and the highest rates in endosymbiosis with Gunnera (14). In symbiosis with both Azolla and Gunnera, the bacterial GS is downregulated, unlike in legume-Rhizobium symbiosis, resulting in up to 40% of fixed nitrogen being released as ammonium. This ammonium is subsequently assimilated by the GS-GOGAT system of the plant host (8, 14) (Fig. 3). In the bryophyte-Nostoc symbioses, up to 80% of fixed nitrogen is excreted to the host as NH_3_, but the mechanisms leading to secretion by the bacterium and incorporation by the plant are still unknown (12) (Fig. 3). In Nostoc-cycad associations, unlike other cyanobacterial symbioses, the GS-GOGAT system of Nostoc is not downregulated, and nitrogen is transferred to the host in the form of citrulline, glutamine, or both, depending on the cycad host (13).

Sugarcane infected with Gluconacetobacter has been reported to acquire up to 60% of its nitrogen from biological nitrogen fixation, although this seems highly varied depending on environmental conditions. Gluconacetobacter loses about 40% of its fixed nitrogen, probably in the form of NH_3_, and this is likely assimilated by the GS-GOGAT pathway of the plant, although this has not yet been demonstrated conclusively (11).

## OTHER NUTRIENTS

Apart from fixed carbon and nitrogen, several other compounds are made available to symbiotic microbes, especially in the case of endosymbionts, which rely on the host for all of their essential nutrients. Phosphorus is essential for metabolism of both the host and microsymbiont but is often the limiting nutrient for nitrogen-fixing plants (85). Iron is an essential component of nitrogenases (Nif, Vnf, and Anf), as well as of leghemoglobin, and appears to be transferred across the symbiosome membrane by a divalent metal ion transporter (86). Sulfur is also an essential component of nitrogenase and must be transferred across the membranes. In L. japonicus the sulfate transporter *Lj*SST1 is essential for nitrogen fixation; knockout mutants are unable to develop functional nodules (87). Other important components of nitrogenases are molybdenum and vanadium. The availability of these trace metals may be critical for the nitrogen cycle of terrestrial ecosystems (88). In the bacterium B. japonicum, for example, molybdenum is transferred by a high-affinity ABC-type ModABC system that is required for efficient nitrogen fixation (89). Finally, rhizobia require cobalt for the biosynthesis of vitamin B_12_, which is involved in the production of exopolysaccharide (90). Transporters of cobalt have been identified in several rhizobia, but none are known for the plant host. Frankia (64) and Nostoc (91) also require cobalt, although its specific role is unknown.

### Oxygen protection.

Biological nitrogen fixation is catalyzed by nitrogenase, a metalloenzyme complex that consists of an iron-protein homodimer and an iron-molybdenum protein heterodimer encoded by the *nifHDK* genes (92). Additional genes in the *nif* operon code for proteins involved in nitrogenase cofactor biosynthesis, electron transport to nitrogenase, regulation, and some proteins with unknown functions (93). The metal clusters in nitrogenase consist of a [4Fe-4S]cubane in NifH, and unique P and FeMo clusters in NifDK. These clusters are inactivated by oxidation of the iron in the metal clusters; thus, nitrogen-fixing microorganisms have evolved various mechanisms to prevent this oxygen poisoning (92, 93). In many diazotrophs, additional operons have been identified as being essential for nitrogenase activity. The *fixABCX* genes are widespread among diazotrophic and nondiazotrophic bacteria (94, 95). Although their exact roles are unknown, they are homologous to electron transfer flavoproteins, ubiquinone oxidoreductase, and ferredoxin and are thought to be involved in electron transport to nitrogenase and possibly in balancing electron flow between nitrogen fixation and other cellular processes, such as respiration.

Microaerobic conditions favorable to nitrogenase are established in legume, Parasponia, and actinorhizal nodules by various mechanisms that include: (i) O_2_ diffusion resistance in outer cell layers of nodules, (ii) binding and transport of O_2_ by leghemoglobins in infected cells of the nodule interior, (iii) restriction of oxygen diffusion into bacteria by external mucilage, and (iv) rapid consumption of O_2_ by bacteria and plant mitochondria. The crucial role of leghemoglobins in *L*. japonicus nodules was demonstrated via RNA interference (RNAi)-mediated repression of leghemoglobin gene expression, which resulted in higher levels of free oxygen, lower ATP-to-ADP ratios, and loss of nitrogenase activity (96). Rhizobia respond to the microaerobic conditions in the nodule through a complex signaling cascade. Low oxygen concentrations activate the oxygen sensor protein FixL, which in turn phosphorylates and thereby activates the transcriptional activator FixJ. The activated FixJ protein induces transcription of *nifA* and *fixK*, and the protein products of these genes induce the transcription of different genes encoding proteins involved in the process of nitrogen fixation. Under these conditions, rhizobia also modify their electron transport chains by expressing the *fixNOPQ* genes that code for a heme-copper *cbb_3_*-type oxidase with high affinity for oxygen (97–99).

Cyanobacteria have evolved several mechanisms to protect nitrogenase from oxygen toxicity. These strategies involve spatial or temporal separation of photosynthesis and nitrogen fixation. Some filamentous cyanobacteria, such as Nostoc (100) and Anabaena (101), develop specialized nonphotosynthetic cells, called heterocysts, where nitrogen fixation occurs. Heterocysts lack the oxygenic photosystem II and are able to maintain microaerophilic conditions by their thick cell walls acting as an oxygen barrier and through active respiration. Other cyanobacteria have the ability to carry out photosynthesis and nitrogen fixation in the same cell. These photosynthetic diazotrophs protect nitrogenase by fixing nitrogen at a time when photosynthesis is depressed, typically at night (102).

There are also examples of plant-associated aerobic heterotrophs with the ability to fix nitrogen, most notably Azotobacter vinelandii, which can grow diazotrophically even under high oxygen tension (103). Various mechanisms for oxygen protection of nitrogenase in A. vinelandii have been proposed. These include a high respiratory rate involving a specialized cytochrome that keeps oxygen levels low inside the cell (103), a protein that binds and protects nitrogenase (but renders it temporarily inactive) under conditions of oxygen stress (104), and the production of an alginate capsule that presumably slows oxygen diffusion into the cell (105). Similar mechanisms have been described for Gluconacetobacter (11, 103, 104).

Frankia spp. fix nitrogen within specialized vesicles that have a high hopanoid content in their membranes. These hopanoids are believed to slow oxygen diffusion across the membrane (106). Additionally, actinorhizal plants, like legumes, fill their nodules with leghemoglobin (107).

Understanding the molecular mechanism of biological nitrogen fixation outside legume-Rhizobium symbiosis could have important agronomic implications. Discoveries and breakthroughs in legume and nonlegume nitrogen fixation provide new insight into ways of manipulating key steps in this process, engineering nitrogen-fixing ability in nonlegume crops, and exploiting the biodiversity of nitrogen-fixing organisms.

## STRATEGIES AND TOOLS FOR ENGINEERING SYMBIOTIC NITROGEN FIXATION IN NONLEGUMES

**FIG 4:**
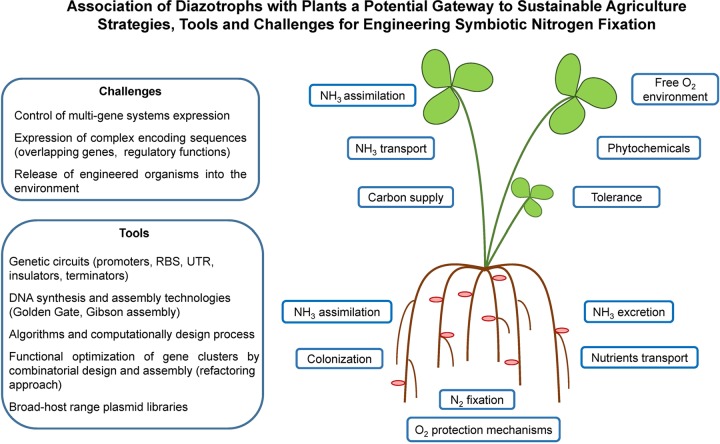
Association of diazotrophs with plants as a potential gateway to sustainable agriculture: strategies, tools, and challenges for engineering symbiotic nitrogen fixation. The availability of nitrogen is one of the principal elements limiting growth and development of crops. Nature solved the nitrogen limitation problem via the evolution of biological nitrogen fixation in diazotrophic bacteria, which reduce atmospheric nitrogen to ammonia (NH_3_) that is subsequently assimilated into biological molecules. Some plants, including most legumes and a few nonlegumes, have evolved the ability to form intimate nitrogen-fixing symbioses with diazotrophs, whereby large populations of diazotrophs are accommodated within living plant cells that provide nutrients to the bacteria in exchange for ammonia produced by nitrogenase. The plant host also protects oxygen-labile nitrogenase from inactivation by reducing free-oxygen. Several factors must be taken into account to engineer a synthetic nitrogen-fixing symbiosis: (i) optimization of the colonization process, (ii) engineering of synthetic *nif* clusters optimized for nitrogen fixation by microsymbionts, (iii) engineering of respiratory protection and O_2_-binding proteins to allow aerobic nitrogen fixation by microsymbionts, (iv) conditional suppression of ammonium assimilation by microsymbionts to ensure nitrogen delivery to plants, (v) ensured effective uptake of ammonium by plant cells, and (vi) optimization of carbon supply from root cells to endosymbiotic bacteria. One of the major limiting factors in engineering symbiotic nitrogen fixation is the challenge to control the expression of multigene systems and complex coding sequences. However, tools have been developed to modularize and control gene expression with precision (promoters, RBS, untranslated region [UTR], insulators, terminators, and broad-host-range plasmids). Nascent computer-aided design algorithms give engineers the ability to create and debug large multigene systems and build synthetic regulation. Intricate designs of large multigene systems are now realizable due to the rise of DNA synthesis and DNA assembly techniques. The use of engineered organisms also raises concerns about the release of genetically modified organisms and their DNA into the environment. Genome-scale engineering approaches can be applied to build safety controls to prevent the survival of genetically modified organisms in the environment and DNA release.

Advances in our understanding of biological nitrogen fixation, coupled with the development of powerful tools for engineering microbes and plants (108, 109), have given rise to different biotechnological approaches to develop cereals and other nonlegume crops that fix nitrogen, namely, the introduction of nitrogen fixation into plants directly, engineering of nonlegume plants to nodulate and establish symbiotic nitrogen fixation, and the development of new tailored associations between nitrogen-fixing microorganisms and crop plants (Fig. 4).

A direct approach to engineering nitrogen fixation in nonlegumes is the introduction of nitrogenase-encoding bacterial *nif* genes into plants. The complexity of nitrogenase biosynthesis and the sensitivity of nitrogenase to oxygen present a significant challenge to implementing this strategy. Extensive genetic and biochemical studies have identified the common core set of genes/gene products required for functional nitrogenase biosynthesis (93). In addition, plastids and mitochondria offer potential subcellular low-oxygen environments to express active nitrogenase in plants, making this engineering strategy feasible (110).

As mentioned previously, most land plants, including cereals, can form arbuscular mycorrhizal associations but are unable to form nitrogen-fixing root nodule symbioses. Although the nitrogen-fixing symbiosis is restricted to legumes, several components of the legume symbiotic signaling (SYM) pathway also play a role in the arbuscular mycorrhizal symbiosis. Since cereals contain the SYM pathway for arbuscular mycorrhizal associations, nodulation could be established in them by engineering the perception of rhizobial signaling molecules to activate this pathway, as well as by engineering its outputs of activation into an oxygen-limited nodule-like root organ for nitrogen fixation.

Engineering synthetic symbiosis in cereal crops by improving nodule-independent association with nitrogen-fixing microorganisms involves the manipulation of both partners to exchange appropriate signals between them to establish successful colonization and nitrogen fixation. In this approach, plants can be engineered to secrete a specialized carbon source that specifically enhances the competitiveness of newly introduced nitrogen-fixing microbes in the rhizosphere. Previous studies have reported the influence of novel nutritional resources in the selection of microbial populations in the rhizosphere (53, 54, 56). Pursuing this biased rhizosphere approach will involve the identification of appropriate plant and bacterial signals, receptors, and target genes to establish a successful artificial symbiosis for nitrogen fixation in cereal crops. Although engineering synthetic symbiosis appears to be less complex than developing endosymbiosis in nonlegume crops, it may be limited in the amount of fixed nitrogen that can be delivered to the crop.

Although all these strategies for transferring nitrogen fixation to crops beyond legumes have complex engineering problems, they have the potential to revolutionize the way cereal crops are grown and provide sustainable food production for the growing global population. It requires collaborative and multidisciplinary efforts involving researchers with diverse skills and expertise to engineer nitrogen-fixing cereals for an affordable eco-friendly agricultural system. Even a small increase in available nitrogen in these self-supported nitrogen-fixing cereals will enable substantial yield increase in the low-input farming systems of developing countries. The positive impact of increasing yield, together with the additional benefit of increasing nitrogen content of the crop with increasing nitrogen applications, was shown in a recent analysis (111). Briefly, with nitrogen fertilizer applications between 0 and 200 kg · ha^−1^, both yield and nitrogen uptake increase substantially. At the highest nitrogen application rate (350 kg · ha^−1^), however, no further yield increase occurs, although further nitrogen uptake is apparent. The inability of the crop to respond to the increased nitrogen at >200 kg · ha^−1^ in terms of increased yield reflects factors other than nitrogen-limited yield, most likely source productivity (111). This source limitation may be intrinsic photosynthetic efficiency or water limitation.

One of the major limiting factors in engineering symbiotic nitrogen fixation in cereals is the availability of a wide range of well-characterized promoter elements in cereals. Use of the same promoter to express multiple genes in transgenic plants can induce gene silencing. A wide range of promoters need to be characterized to drive an equivalent expression of several transgenes in the same cells. Another immediate barrier to engineering nitrogen-fixing capability in cereal crops is the construction of large multigene synthetic cassettes. However, new DNA assembly strategies, such as Golden Gate (112) and Gibson assembly (113), enable straightforward and time-efficient development of large numbers of multigene constructs. Anticipating possible bottlenecks in cereal transformation, it is necessary to develop highly efficient transformation methods as well as high-throughput transient gene expression systems for these crops to reduce development time. The possibility to transform the model cereal Setaria viridis by floral/spike-dip transformation offers exciting avenues to accelerate cereal engineering (114).

Although engineering new more-robust microbe-mediated nitrogen fixation associations is considered to be more tractable, there are several challenges unique to the problem of designing symbiotic plant-microbe interactions that facilitate nitrogen delivery to cereal crops. The first is that nitrogen fixation requires many genes that are tightly regulated in their native host and are sensitive to environmental conditions that are not desirable in agriculture (e.g., repressed by high ammonia) (97, 115). Overcoming this issue requires either transferring the pathway from one organism to another or unsilencing a cluster in a native host (116–118). Both of these are impeded by the fact that the pathway is very sensitive to small changes in gene expression (119), and the regulatory control in many organisms is not well characterized. In addition, the microbe needs to establish a symbiotic relationship with the crop, which either requires engineering the plant or using endophytes, for which there may be few genetic tools (e.g., transposon mutagenesis). The ability to stringently control this association and eliminate escape mutants and DNA release to the environment will be another aspect to take into consideration when developing strategies and tools for engineering symbiotic nitrogen fixation, especially with regard to regulatory and environmental issues surrounding the release of genetically modified microorganisms.

Building a synthetic multigene system with these parts is now relatively straightforward (Fig. 4). However, it is more complicated when working with a gene cluster obtained from nature in which many genes are already under native control. The genes within these clusters often have complex encoding, including overlapping genes and regulatory functions (120). Because of this, it is difficult to change a part, such as a ribosome biding site (RBS), without impacting many other aspects of the system. To address this, Temme et al. (119) applied the process of refactoring (121, 122) to the 16-gene *nif* cluster from Klebsiella oxytoca, which modularized the gene cluster into a set of well-characterized genetic parts. This system can be used as a platform for large-scale part substitutions that facilitate the swapping of regulation to one which will function in a new host (123). Refactoring has also been shown to be valuable in eliminating the response to signals that repress the native *nif* cluster, including ammonia and O_2_ (119, 124).

Prior to the emergence of DNA synthesis, the process of genetic engineering largely involved small-scale cloning steps focused on combining natural DNA sequences. The last decade has seen tremendous advances in DNA synthesis that lowered the cost and turnaround time while increasing the fidelity and size of DNA that can be ordered (125). This has led to larger sequences and libraries being constructed, including the first biosynthetic gene cluster (126), the *nif* cluster from Klebsiella (119), and even entire chromosomes and prokaryotic genomes (127, 128). There is even an effort to synthesize an entire eukaryotic genome (Yeast 2.0). Clearly, refined knowledge of the determinants and requirements of biological nitrogen fixation has converged with the advancement in the development of synthetic biology technologies to make a challenging problem of engineering of new plant-microbe nitrogen fixing associations a tractable venture.

## CONCLUSION

The creation of artificial symbioses or associations between diazotrophs and crops is a primary goal in agriculture to reduce the demand for chemical nitrogen fertilizers. Improved understanding will lead to (i) more-sustainable exploitation of the biodiversity of nitrogen-fixing organisms, and (ii) the transfer of biological nitrogen fixation capacities to major nonlegume crops. Since much of the basic work, major breakthroughs, and discoveries have been done on legumes, strategies to expand the genetic capacity to fix nitrogen in symbiotic relationships are currently based on that knowledge. Recent advances in the understanding of endosymbiotic, associative, and endophytic nitrogen fixation with legumes and nonlegume plants may lead to novel avenues for engineering nonlegume nitrogen-fixing crops.
